# Oxidative Stress Mitigation by Chitosan Nanoparticles in Durum Wheat Also Affects Phytochemicals and Technological Quality of Bran and Semolina

**DOI:** 10.3390/plants11152021

**Published:** 2022-08-03

**Authors:** Valentina Picchi, Antonella Calzone, Serena Gobbi, Sara Paccani, Roberto Lo Scalzo, Alessandra Marti, Franco Faoro

**Affiliations:** 1 CREA Research Centre for Engineering and Agro-Food Processing, via G. Venezian 26, 20133 Milano, Italy; antonella.calzone@crea.gov.it (A.C.); sara.paccani@crea.gov.it (S.P.); roberto.loscalzo@crea.gov.it (R.L.S.); 2 Department of Food, Environmental and Nutritional Sciences, Università Degli Studi di Milano, Via Celoria 2, 20133 Milano, Italy; serena.gobbi@unimi.it (S.G.); alessandra.marti@unimi.it (A.M.); 3 Department of Agricultural and Environmental Sciences, Università Degli Studi di Milano, Via Celoria 2, 20133 Milano, Italy

**Keywords:** chitosan, nanoparticles, oxidative stress, durum wheat, polyphenols, tocols, semolina

## Abstract

In our previous work, durum wheat cv. Fabulis was grown over two consecutive seasons (2016–2017 and 2017–2018) in an experimental field in the north of Italy. With the aim of mitigating oxidative stress, plants were subjected to four treatments (deionized water, CHT 0.05 mg/mL, CHT-NPs, and CHT-NPs-NAC) three times during the experiment. Chitosan nanoparticles (CHT-NPs) reduced symptom severity on wheat leaves and positively influenced the final grain yield. The present work aimed at investigating whether CHT treatments and particularly N-acetyl cysteine (NAC)-loaded or -unloaded CHT-NPs, while triggering plant defense mechanisms, might also vary the nutritional and technological quality of grains. For this purpose, the grains harvested from the previous experiment were analyzed for their content in phytochemicals and for their technological properties. The results showed that CHT increased the polyphenol and tocopherol content and the reducing capacity of bran and semolina, even if the positive effect of the nano-formulation remained still unclear and slightly varied between the two years of cultivation. The positive effect against oxidative stress induced by the chitosan treatments was more evident in the preservation of both the starch pasting properties and gluten aggregation capacity, indicating that the overall technological quality of semolina was maintained. Our data confirm the role of chitosan as an elicitor of the antioxidant defense system in wheat also at the grain level.

## 1. Introduction

Wheat is the most widely cultivated crop in the world, representing the staple food for an estimated 35% of the world’s population [1], thus being crucial to the food and nutrition security of populations worldwide. A high number of studies have shown that the impact of increasing air pollution on wheat production should not be underestimated. Among air pollutants, ozone (O_3_) is a major threat to wheat [2]. The concentrations of this gaseous pollutant in the northern hemisphere have increased by 40% since the preindustrial era, and, during the past 22 years, median O_3_ values have increased in the free troposphere by 2 nmol mol^−1^ per decade (5% per decade) [3]. O_3_ is a phytotoxic gas that enters plants primarily through the stomatal pores during photosynthetic gas exchange and rapidly dissolves in the aqueous apoplast, producing reactive oxygen species (ROS). O_3_ and O_3_-derived ROS can cause direct oxidative damage, such as lipid peroxidation, chlorophyll degradation, protein oxidation, damage to nucleic acids, and destruction of cell membranes [4]. O_3_ reduces the photosynthetic rate by impairing Rubisco activity, inducing stomatal closure, and altering carbon allocation, thus finally leading to yield losses [5,6]. In wheat, elevated O_3_ accelerates leaf senescence, and the greater damage occurs under high chlorophyll content and light-saturated photosynthetic conditions, i.e., during the late period of the growing season (after anthesis and during grain filling) [7,8,9]. A meta-analytic evaluation of the peer-reviewed literature by Feng et al. [10] reported yield reductions in wheat in the range of 24–34 percent due to elevated O_3_ (average 7–8 h O_3_ concentration of 72 ppb). From the same meta-analysis, the authors indicated that the most important yield component responsible for grain yield reduction was the decrease in individual grain weight, although both ears per plant and grains per ear showed significant decreases at elevated O_3_ concentrations.

Unlike the grain yield, much less attention has been paid to O_3_ effects on the wheat grain quality and, particularly, on nutritional values. In fact, most of the literature reports mainly data about protein (yield and concentration) and starch content. In the study by Broberg et al. [11], the authors synthesized the effects of O_3_ on wheat quality based on 42 experiments performed all over the world and showed that elevated O_3_ improved the quality of wheat grain concerning the concentration of protein and minerals but reduced their amount accumulated per unit area. Crops under stress, e.g., by O_3_, seem to maintain nitrogen uptake, although reduced, to a larger extent than biomass accumulation, thus determining the final reduction in protein yield [12]. This effect may have potentially important consequences for human nutrition. However, as regards the O_3_ positive effect on protein concentration, Pleijel et al. [13] did not observe the same result when comparing non-filtered (NF) and charcoal-filtered (CF) grown wheat plants, suggesting that the improved grain quality due to a higher protein concentration is probably observed only in the case of elevated O_3_ concentrations, and it is of limited importance at present ambient O_3_ levels. In their work, Piikki et al. [14] reported that O_3_ also tended to affect some quality variables towards more mature levels (e.g., increased Zéleny value and Hagberg falling number), indicating that O_3_-exposed plants may have experienced early senescence. O_3_ seems to positively affect the baking properties of wheat, as a consequence of the enhanced (gluten) protein concentration and because the O_3_ promotes wheat premature senescence [11,14]. Late ripening often can result in high α-amylase activity in the grain, lowering its quality and, in the worst case, promoting germination of the grains before harvest [15]. A strong negative relationship between starch and O_3_ concentration was found, which was probably due to the very small and/or malformed kernels that are formed under O_3_ exposure [11]. 

Amelioration of O_3_ effects on crops can be the most appropriate method for preventing yield and quality losses. Several studies have shown that chemical protectants such as antioxidants, pesticides, physical barriers, antiozonants (e.g., ethylenediurea), and plant growth regulators might be interesting approaches to minimize the damages caused by O_3_ [16]. Among others, green synthesis of nanoparticles (NPs) seems to be a very effective method in developing a rapid, non-toxic, clean, and eco-friendly approach. Recently, Kannaujia et al. [17] explored the efficacy of biogenic silver nanoparticles against the deleterious effects of O_3_ on wheat. They found that, at specific concentrations, they act as ozone protectants, as they were able to enhance antioxidant metabolites and antioxidant enzymes, finally leading to increased grain yields. The antioxidants of the ASA–GSH cycle, i.e., ASA and GSH, represent the main plant defense strategy against oxidative damage caused by ROS under elevated O_3_ and regulate oxidative damage by scavenging ROS [18,19]. Higher ASA pools are responsible for better O_3_ tolerance in durum wheat cultivars, and the concentrations of antioxidants vary with growth stages [20,21]. Recently, there has been increasing interest toward chitosan (CHT) applications in agriculture (as NPs and not), for its antioxidant [22], anti-microbial [23], bioactive [24], and non-toxic [25] properties, besides those as a biotic elicitor. Through foliar or soil application, it can induce stress tolerance and improve plant performance by activating several stress-related enzymes and signaling [26,27]. In our previous experiment [28], we showed the capacity of the foliar application of chitosan nanoparticles (CHT-NPs)—both loaded and unloaded with the antioxidant N-acetyl cysteine (NAC)—to increase the leaf antioxidant potential (particularly ascorbate content) of field-grown durum wheat. The findings indicated that CHT-NPs had a positive effect on the final grain yield, particularly increasing the 1000-grain weight. Based on these promising results, we wanted to assess whether CHT treatments, and particularly NPs, while triggering plant defense mechanisms, might also vary the nutritional and technological quality of the grains. Therefore, the focus of the present paper was to verify whether the protective effect of CHT-NPs observed in the plants was extended also to seeds, improving grain quality both from a nutritional and technological point of view, which are indeed important aspects never explored before. To this aim, the phytochemical content and reducing capacity of the grains harvested in the previous experiments were analyzed, together with the technological properties of semolina.

## 2. Results

### 2.1. Effect of CHT Nanoparticles on the Nutritional Quality of Durum Wheat Bran and Semolina

#### 2.1.1. Phenolic Acid Content

The main component detected in both bran and semolina was ferulic acid, which reached around 80% of the total, followed by 8% sinapic acid. The amount of the identified compounds is shown in Table 1, while, in Figure S1, we present a typical chromatographic profile obtained for bran.

**Figure 1:**
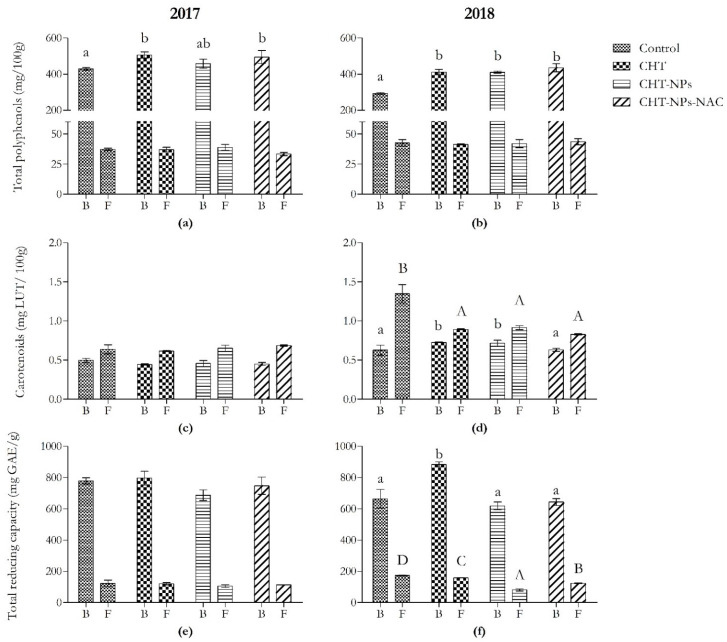
Content of total polyphenols, carotenoids, and total reducing capacity in bran and semolina obtained from *Triticum durum* (cv. Fabulis) in 2017 (**a**,**c**,**e**) and 2018 (**b**,**d**,**f**), untreated (Ctrl) or treated with CHT, CHT-NPs, or CHT-NPs-NAC. Bars represent mean ± standard deviation (*n* = 3). Different uppercase and lowercase letters indicate significant differences among treatments in bran and semolina, respectively, according to Tukey’s post-hoc test.

As shown in Figure 1a,b, total polyphenols increased only in bran samples. In the first year, they were +17% higher than controls in CHT and CHT-NPs-NAC samples. These results were probably related to the significant variations observed in the content of some phenolic acids, namely ferulic acid, vanillic acid, and syringic and sinapic acid (Table 1). In particular, CHT and CHT-NPs-NAC applications induced a significant increase in ferulic acid (+22%) and syringic acid (+35%), whereas no significant differences were observed in CHT-NPs-treated plants. Moreover, vanillic and sinapic acid increased in all treatments. The latter increased in the same way in all treatments. Vanillic acid showed the highest increase in the CHT-NPs-NAC treatment (+61%), followed by the CHT (+43%) and CHT-NPs (+15%) ones. On the contrary, semolina samples did not show any significant variations in the first year (except for p-coumaric acid, which decreased by −25% in CHT-NPs-NAC samples).

In 2018, all treatments induced an increment in total polyphenols in bran samples (+41% compared to the control). A similar trend was observed for ferulic, vanillic, and sinapic acid, which increased in all treatments. The latter showed the highest increase in the CHT-NPs-NAC treatment (+54%), followed by the CHT and CHT-NPs ones (+29%), whereas ferulic and vanillic acid increased in the same way (+37% versus controls). Moreover, syringic acid showed an increment only in the CHT and CHT-NPs-NAC treatments (+44%). In semolina, only the CHT-NPs-NAC treatment induced an increase in both syringic (+26%) and vanillic acid (+104%), whereas the latter increased even in CHT samples (+29%).

#### 2.1.2. Tocopherol and Tocotrienol Content

As shown in Table 2, in 2017, both CHT and CHT-NPs-NAC induced a significant increase in total tocopherol (T) content in bran (+57.9 and +92.6%, respectively), while tocotrienols (T3) were increased only in semolina treated with CHT-NPs-NAC (+25%).

In particular, the α-T amount was, respectively, +47 and +84% higher than controls in CHT and CHT-NPs-NAC treatments. Although no significant differences were observed in terms of total T3, (β + γ)-T3 increased in CHT samples (+20%) and decreased in CHT-NPs ones (−15%) compared to the controls. In CHT-NPs-NAC-treated semolina samples, the high content of total T (+21%) and total T3 (+26%) was reflected in the α-T and (β + γ)-T3 fractions increasing by +39% and +26%. Nevertheless, no significant differences were observed in the other two treatments, except for CHT-NPs, showing an increase of +35% in (β + γ)-T3 content (Table 2).

Unlike 2017, in the second year, CHT and CHT-NPs applications increased the tocols content in bran (+11 and +24, respectively) and semolina (+18 and +20%, respectively) . In bran, these results were probably related to the significant variations observed in T and T3 content (Table 2). The total T amount increased by +9% in both treatments; in particular, (β + γ)-T was +20% higher than controls only in the CHT treatment. Regarding total T3, the highest level was observed in CHT-NPs samples (+24%), probably due to increased amounts of α-T3 and (β + γ)-T3 (+19 and +24%, respectively). Moreover, in CHT plants, (β + γ)-T3 showed an increase of +10% compared to the controls. 

Similarly, CHT and CHT-NPs applications induced an increment in total T (+11%) and T3 (+29%) content in semolina samples. In particular, (β + γ)-T increased by +20% in the CHT treatment and α-T by +31% in the CHT-NPs ones. Indeed, both treatments induced the same increment in (β + γ)-T3 content (+29%), and an increase in α-T3 was also observed only in CHT-NPs samples (+59%). 

The opposite trend was observed in CHT-NPs-NAC samples. Although no significant differences were observed in terms of tocols content in bran samples , α-T content decreased by −50%, entailing a reduction of −29% in total T. On the contrary, in semolina samples, a slight reduction in tocols (−7%) was associated with a decrease of −53% in α-T content (leading to a reduction in total T of −25%).

#### 2.1.3. Total Carotenoid Content

As shown in Figure 1c,d, carotenoid levels changed only in 2018 in both bran and semolina samples, but with a different trend. On the one hand, CHT and CHT-NPs treatments induced an increment in these compounds (+15%) in bran samples, whereas no significant differences were observed in CHT-NPs-NAC ones. On the other hand, all treatments caused a reduction in carotenoid content (−35% versus controls) in semolina samples.

#### 2.1.4. Total Reducing Capacity

Variations in total reducing capacity were observed only in 2018 (Figure 1e,f). It increased only in bran samples treated with CHT (+33%), whereas no significant differences were observed in the other two treatments. On the contrary, semolina samples showed a decrease in total reducing capacity in all treatments: −9% in CHT; −53% in CHT-NPs, and −28% in CHT-NPs-NAC versus controls.

### 2.2. Effect of CHT Nanoparticles on Technological Properties of Durum Wheat Semolina

#### 2.2.1. Total Starch, Damaged Starch, and Pasting Properties

None of the considered treatments significantly affected either the total starch or damaged starch content (Table 3).

Table 3 also reports the main indices used to evaluate the starch pasting properties. The test measures the changes in the viscosity of a suspension of starch and water as affected by heating and cooling under controlled conditions. Native starch is insoluble in cold water, but as the temperature increases, starch granules absorb water and swell, increasing the viscosity of the system. This phenomenon is known as gelatinization, and the point at which an initial increase in viscosity occurs corresponds to the beginning of gelatinization. When starch granules reach the maximum extent of molecular disorder, the maximum viscosity is registered by the device. After this point, starch granules break, the amylose and amylopectin fractions exit the granule, and a decrease in viscosity is registered. The drop in viscosity is known as “breakdown” and it provides information about the starch’s stability to both heating and shear stress. During cooling, gelatinized starch undergoes structural reorganization, with the formation of an ordered structure; this phenomenon is known as retrogradation and increases viscosity. The extent of retrogradation is given by the setback value, which is calculated as the difference between the final viscosity and the viscosity reached at the end of the holding period. The applied treatments did not significantly affect the starch pasting properties of semolina (Table 3), suggesting similar behavior of samples during processing that involves heating and cooling steps (such as pasta, for example). Although the one-way ANOVA was significant for the temperature at the beginning of gelatinization, all chitosan treatments did not show significant differences compared to the controls. The only significant difference was observed between CHT-NPs and CHT-NPs-NAC samples grown in 2018 (Table 3). 

#### 2.2.2. Proteins, Thiol Content, and Gluten Aggregation Capacity

In semolina, total thiol content showed the opposite trend between the two years (Table 4). In the first year, all treatments induced a decrease of –18% compared to controls, while quite the opposite in the second year—an increment of +10%—was observed in all treatments. However, such changes were not significant, as also for total protein content.

In 2017, the applied treatments did not significantly affect the gluten aggregation properties of semolina (Table 4), suggesting similar behavior of samples during processing that involves gluten formation (such as pasta, for example). On the other hand, in 2018, a significant difference was observed in the maximum torque between CHT-NPs and CHT-NPs-NAC compared to control samples (Table 4).

## 3. Discussion

The role of chitosan as an abiotic elicitor was widely reported in several works [29,30], especially highlighting its capacity to activate plant responses against abiotic (e.g., O_3_) and biotic (e.g., Fusarium head blight disease) stress [31]. In our previous work [28], we reported that CHT-NPs applications increased the leaf antioxidant pool, mitigating O_3_’s negative impact on wheat plants. Moreover, the significant increase in the 1000-grain weight evidenced a positive impact of CHT-NPs on the wheat grain yield. Given the above, this work aimed to investigate, for the first time, the effects of CHT and NAC-loaded or -unloaded CHT-NPs on the technological and nutritional quality of the wheat grain, such as polyphenols and vitamin E content, at the bran and semolina level. 

Although the experiment was conducted in the same way, in the same parcel, and with the same variety, different trends were observed between the two years. It is worth noting that environmental conditions (e.g., temperature, rainfall, etc.), crop year, and their interaction had significant effects on antioxidant content (e.g., flavonoids, phenolic acids, etc.) [32]. De Santis et al. [33] documented how environmental changes influenced the antioxidant profile of wheat grain. In particular, it was observed that years with lower levels of rainfall induced an increment in lipophilic antioxidants (such as tocols), whereas polyphenols were much higher in crop seasons showing lower temperatures in the 30 days before harvesting. Similarly, in our experiment [28], 2017 was characterized by higher temperatures (especially at the ripening stage) and lower total rainfall (especially in the booting stage) than 2018, which may have been responsible for the higher constitutive levels of phenolic acids and tocols measured in 2017 compared to 2018. 

As explained in the Introduction, O_3_’s negative impact on wheat plants is widely reported, affecting starch and mineral content at the grain level [34]. As a matter of fact, in our experiment, the O_3_ critical level (3000 ppb.h for agricultural crops) was reached two weeks earlier in 2018 than in 2017, thus determining an increase in the plants’ exposure to the pollutant during the early ripening stage. Nevertheless, the grain quality of chitosan-treated samples did not seem affected by the oxidative stress induced by the longer O_3_ exposure. Higher variations in terms of antioxidant compounds were observed in 2018 than in 2017, both at bran and semolina level, indicating stronger activation of the defense system enhanced by chitosan treatments. It is worth noting that changes in vitamin E components at the bran level were reflected also in semolina in all chitosan treatments. The decrease in reducing capacity in chitosan-treated semolina samples could reflect antioxidant activity against the ongoing oxidative stress induced by O_3_ to counteract its negative effect at the endosperm level. Fatima et al. [21] reported increased levels of thiols (ROS-detoxifying compounds) against O_3_ stress in *Triticum aestivum* cultivars, suggesting thiols as a biomarker of O_3_ tolerance. Besides their antioxidant activity, thiols also affect the rheological properties of dough, both by lowering the number of intermolecular disulfide bonds and/or increasing the rate of their exchange [35]. In our experiment, thiol content in semolina did not significantly vary depending on CHT treatments. On the other hand, the main differences were observed in the phenylpropanoid pathway and vitamin E components, mainly in the outermost wheat layers, which are rich in these components [36]. Bran quality, in fact, was positively influenced in both years, with increased levels of (i) the most important phenolic acids and of (ii) (β + γ)-T3 and -T, which have greater antioxidant activity compared to α-T3 and α-T [37]. At the semolina level, positive effects were observed only in 2018, with an increment in tocols, the same as observed in bran, and vanillic acid. The latter is one of the most important phenolic acids in wheat and, besides its antioxidant properties, it is characterized by a strong flavor, conferring peculiar sensory properties to the final product [38]. Chandra et al. [39] reported higher efficiency in plant defense modulation of CHT-NPs than CHT, due to its better bio-accessibility. These results are in accordance with what was observed at the leaf level but partially disagree with those observed at the grain level, highlighting different responses among tissues within the same plant. CHT-NPs’ major effects were observed only in the second year, with an increment in phenolic acids and tocotrienols in bran. Similarly, an increase in tocotrienols was observed in semolina, in addition to syringic acid, one of the most important benzoic acid derivatives in wheat grain.

As observed for CHT, CHT-NPs-NAC applications induced positive effects on grain quality in both years, affecting both nutritional and technological properties. Colak et al. [40] reported that NAC applications enhance the antioxidant defense system in wheat by promoting phenolic acid synthesis; likewise, our results showed an increased level of ferulic, vanillic, syringic, and sinapic acids in both years. Unlike the other two treatments, the CHT-NPs-NAC application was the only one to enhance semolina quality in both years, but with different trends, highlighting how the final product may be differently affected depending on the cultivation year [41]. The first year was characterized by increased levels of both tocotrienols and tocopherols, whereas the second one by two benzoic and cinnamic acid derivatives (i.e., vanillic and syringic acids). The enhanced synthesis of tocols, observed only in 2017, could be related to the higher temperatures than in 2018 at the ripening stage. Yoshida et al. [42], studying the chemical and physical behavior of tocols in membranes, explained the capacity of tocopherols to increase the rigidity of membranes, which is compromised under high temperatures. 

A separate comment should be made regarding carotenoid content, which showed different variations in bran and semolina only in the second year. Several works reported how chitosan applications induced an increment in photosynthetic pigments (accessory and not) in many species (e.g., *Triticum aestivum*, *Stevia rebaudiana*, *Oryza sativa,* etc.) [43]. Similarly, our results showed that NAC-unloaded CHT applications increased the content of these accessory pigments in bran, contributing to yellowness [44]. Conversely, all treatments decreased carotenoid content in semolina, probably due to the activation of lipoxygenase at the endosperm level by chitosan [45], resulting in the oxidation of these antioxidant compounds [46]. 

The protective effect against O_3_ stress induced by the chitosan treatments was reflected in the technological properties of semolina by preserving pasting properties and low damaged starch content. The latter represents the amount of starch that has been physically damaged during the milling process. In the case of durum wheat semolina, low levels of damaged starch are preferred to ensure high pasta quality, especially in terms of low heat damage [47]. The gluten aggregation properties of semolina samples were evaluated by the GlutoPeak test. During the test, semolina and water are mixed at a very high speed (2750 rpm): as the gluten is formed, the device registers an increase in consistency till a maximum value (i.e., maximum consistency at a certain time, called peak maximum time), which corresponds to the maximum gluten aggregation. Although we observed some differences between the two years of the experiment, the data obtained in 2018 indicate a higher maximum torque in CHT-NPs and CHT-NPs-NAC compared to control samples. Generally, strong semolina shows high values for both maximum torque and peak maximum time [48]. This result might indicate that CHT-NPs and CHT-NPs-NAC tended to induce a technological enhancement in semolina. Although the total protein content did not change, fluctuations in their composition (ratio of gliadins to glutenins) cannot be ruled out, as the addition of CHT-NPs and CHT-NPs-NAC resulted in an increase in consistency at the maximum aggregation of the gluten aggregation (maximum torque) compared to the control samples (*p* < 0.05). Indeed, Marti et al. [49] demonstrated a correlation between gliadin content and maximum torque.

To sum up, the combination of long O_3_ stress exposure, high temperatures, and low rainfall during the ripening stage influenced the biosynthesis of the major antioxidants of wheat grain, which are involved in plant defense against environmental stress. Our data confirmed what was observed in the previous work: the role of chitosan as an elicitor of the antioxidant defense system in wheat both at leaf and grain levels. Chitosan treatments had a positive effect on the nutritional quality of the processed product (i.e., semolina), which is usually poor in antioxidant compounds compared to bran [50]. In particular, all chitosan treatments seemed to have a positive effect on benzoic acid derivatives—in particular, vanillic and/or syringic acids. CHT and CHT-NPs-NAC treatments were still able to exert their positive effects, along with different environmental conditions. In addition, the NAC-loaded CHT treatment positively affected semolina quality over time, with environmental-dependent variations toward the synthesis of either phenolic acids or tocols, whereas the NAC-unloaded CHT treatments induced the synthesis of both fractions. Although in different ways and under different conditions, all treatments positively affected the content of vitamin E compounds and phenolic acids, thereby improving the quality of the final product. It is worth noting that increased levels of these antioxidants (i) enhance the plant defense system against abiotic and biotic stress [51] and (ii) improve grain quality (functional and sensory level), both as animal feed and for human consumption [52]. Several studies showed how chitosan treatments (both as NPs and non-NPs) can alleviate the negative impact of various stresses by acting both at the gene expression level and at the biochemical level. They can induce stress tolerance and improve plant performance by changing primary metabolites, hormone signaling, antioxidant activity, secondary metabolites, and membrane permeability, leading to improved photosynthesis and growth [26]. Further studies are required to investigate and deepen the specific processes involved in/influenced by CHT applications at the transcriptional level in relation to the effects mentioned above in durum wheat grain.

## 4. Materials and Methods

### 4.1. Experimental Design and Sample Preparation

Durum wheat (*Triticum durum* Desf. cv. Fabulis) was grown over two consecutive seasons (2016–2017 and 2017–2018) in an experimental field situated near the city of Voghera (44°59′43″ N; 9°2′56″ E), as detailed in Picchi et al. [28]. Cultivar Fabulis was chosen because of its high sensitivity to oxidative stress, as assessed by visual leaf symptoms during the latest three years of field observations. These observations were extended to numerous wheat cultivars in the same growing area during the last two decades [53]. During the experiment, plants were subjected three times to four treatments (deionized water, CHT 0.05 mg/mL, CHT-NPs, and CHT-NPs-NAC), with the aim of mitigating oxidative stress in wheat. Ambient ozone concentration was continuously monitored by a nearby weather station, as well as temperature and rainfall. The critical level of 3.000 ppb.h for agricultural crops was exceeded on the 7th of June in 2017 and the 17th of June in 2018. More details about the experimental plan are reported in Picchi et al. [28]. At harvest, grains were milled into semolina, separating the bran using a mill, namely the Labormill 4RB (BONA srl, Monza, Italy). Bran and semolina were stored at −20 °C until analysis.

### 4.2. Analysis of Polyphenols

Polyphenols were extracted from semolina and bran following Mazzoncini et al.’s [54] method, with slight modifications. Samples (0.2 g) were alkaline hydrolyzed under nitrogen with 4 N NaOH (5 mL) and then 0.2 mL of 5% ascorbic acid was added to prevent the degradation of phenolic acids. Samples were acidified using 6 N HCl to achieve a pH value of 1–2. The mixture was extracted twice with 10 mL of ethyl acetate and 1 mL of 20% (*w/v*) of NaCl. The organic phase was collected and evaporated to dryness in a centrifugal evaporator. Before analysis, the residue was re-dissolved in methanol: 0.5% formic acid (ratio 80:20) to a final volume of 2 mL. The phenolic composition was evaluated by HPLC-DAD, using a Jasco system equipped with a diode array detector, MD-910 (Jasco, Mary’s Court, Easton, MD, USA). Then, 10 µL of undiluted extract was injected into an HPLC system and the separation was performed with a Kinetex EVO C18 100 Å column (250 × 4.6 mm, 5 µm) at 45 °C with a flow rate of 0.6 mL/min. The mobile phase consisted of 0.5% (*v/v*) formic acid:methanol (ratio 95:5) (solvent A) and methanol:H_2_O:acetic acid (95:2.5:2.5) (solvent B). The gradient (A/B) was: 92/8 from 0 to 5 min, from 92/8 to 77/23 in 10 min, 77/23 for 5 min, from 77/23 to 60/40 in 15 min, 60/40 for 5 min, from 60/40 to 40/60 in 5 min, 40/60 for 5 min, from 40/60 to 92/8 in 5 min, 92/8 for 10 min. The total analysis run was 65 min. Phenolic compounds were monitored at 280 nm for benzoic acid derivatives and 320 nm for cinnamic acid derivatives. The peaks were identified both by direct comparison with commercial standards and by their spectral and chromatographic properties. Quantification was based on using calibration curves of external standards: vanillic acid, syringic acid, caffeic acid, p-coumaric acid, sinapic acid (range 0.005–0.05 mg/mL), and ferulic acid (range 0.01–0.2 mg/mL). Results were expressed in mg for 100 g of sample.

### 4.3. Analysis of Tocopherols, Tocotrienols, and Total Carotenoids

Tocopherols and tocotrienols were determined following Lampi et al.’s [55] method. Samples (0.5 g) were saponified with 5 mL of 0.5 M KOH (in EtOH) and 0.2 mL of 5% (*w/v*) of ascorbic acid in a water bath at 80 °C for 15 min. After cooling, tocols were extracted twice with 5 mL of hexane:acetone (ratio 80:20), 100 µL of 1% (*w/v* in MeOH) butylated hydroxytoluene (BHT), and 5 mL of 20% (*w/v*) of NaCl. Samples were sonicated for 15 min and centrifuged at 10,000× *g* for 5 min. The organic phase was collected and evaporated to dryness in a centrifugal evaporator, as reported above. The residue was dissolved in 1 mL of EtOH:acetone (1:1) with 0.2% BHT. Then, samples were sonicated for 15 min and centrifuged at 10,000× *g* for 5 min before HPLC analysis.

Tocols characterization was performed using a Jasco system equipped with a fluorometric detector (FB 1520, Jasco, Tokyo, Japan). First, 30 µL of undiluted extract was injected into an HPLC system and the separation was performed using a YMC C30 column (250 × 4.6 mm, 3 µm) at 40 °C, with a flow rate of 0.55 mL/min. The mobile phase contained metil-t-butyl ether (solvent A) and acetonitrile:MeOH (ratio 90:10) (solvent B). The gradient (A/B) was: 95/5 from 0 to 1 min, from 95/5 to 85/15 in 9 min, 85/15 for 10 min, from 85/15 to 95/5 in 4 min, and 95/5 for 6 min. The total analysis run was 30 min. For identification and quantification, commercial standards of α- and γ-tocopherols were used for tocopherols (range 5–35 µg/mL), while Annatto (*Bixa orellana* L.) seed powder was used for tocotrienols (range 1–40 µg/mL). Results were expressed in mg for 100 g of sample.

Total carotenoid content was determined spectrophotometrically by measuring the absorbance at 446 nm, using the same extracts as for tocols determination. Lutein was used for quantification (molar extinction coefficient 145,000 L mol^−1^ cm^−1^) [56]. Results were expressed as lutein equivalents (LUE) per 100 g of samples. 

### 4.4. Analysis of Total Reducing Capacity

The same extract used for phenol composition was used for the determination of total reducing capacity, following the Folin–Ciocalteu colorimetric assay [57]. First, 100 µL of diluted extract (1:1 with MeOH: formic acid, ratio 80:20) was added to 2 mL of deionized water and 500 µL of Folin–Ciocalteu reagent. After 3 min, 1.5 mL of 10% Na_2_CO_3_ was added to the mixture and it was dark-incubated at room temperature for 2 h. The absorbance of samples was measured at 730 nm using a UV–Vis spectrophotometer (UVIDEC-320, Jasco, Tokyo, Japan), and the concentrations were determined using a calibration curve prepared with gallic acid (GAE) (range 0.02–0.5 mg/mL). Results were expressed as mg GAE equivalents per g of samples. 

### 4.5. Starch Analyses and Pasting Properties on Durum Wheat Semolina

Total and damaged starch was measured in duplicate according to the standard method AACC 76-13.01 and 76-31.01, respectively [58]. Pasting properties were evaluated using a Micro Visco-Amylo-Graph, MVAG (Brabender GmbH., Duisburg, Germany). First, 12 g of semolina was dispersed in 100 mL of distilled water, scaling both sample and water weight on a 14% moisture basis. The suspensions were subjected to the following temperature profile: heating from 30 up to 95 °C, holding at 95 °C for 20 min, cooling from 95 to 30 °C, and holding at 30 °C for 1 min. A heating/cooling rate of 3 °C/min was used. Each sample was analyzed in duplicate.

### 4.6. Protein and Total Thiol Analyses

Protein content was determined in triplicate using the standard method ICC 167. The content of total thiols was determined following the method of Fierens et al. [59] after reaction with 5,5′-dithio-bis (2-nitrobenzoic acid) (DTNB). Semolina samples (0.5 g) were mixed with 5 mL of 50 mM Na/P buffer (pH 8), containing 1 mM ethylene diamine tetra-acetate to prevent thiol oxidation. The suspension was vortexed for 30 s, shaken (100 times/min) for 20 min at room temperature, and centrifuged at 15,000× *g* for 20 min at 4 °C. After filtration (0.45 µm), 1 mL of extract was mixed with 100 µL of 0.1% (*w/v*) DTNB and kept in the dark for 70 min at room temperature. Finally, the absorbance was determined spectrophotometrically at 412 nm. Total thiol content was quantified using a reduced glutathione (GSH) calibration curve (0.04–0.004 mg/mL) and expressed as mg GSH per 100 g of sample.

### 4.7. Gluten Aggregation Properties

Gluten aggregation properties were assessed using the GlutoPeak^®^ (Brabender GmbH and Co KG, Duisburg, Germany). Semolina (9 g) was dispersed in distilled water (10 g; adjusting the quantity of both sample and water on a 14% basis) and mixed at 2750 rpm, keeping the bowl temperature at 35 °C. The analysis was carried out in duplicate.

### 4.8. Statistical Analysis

Data with normal distribution were analyzed following the Shapiro–Wilk test. The effects of the treatments were determined using a two-way ANOVA test, and significant differences among groups were tested by Tukey’s HSD post-hoc test. Statistical analyses were performed with JMP 13 Pro (SAS Institute, Cary, NC, USA). Statistical significance was considered for *p* ≤ 0.05.

## 5. Conclusions

The present work shows that the use of chitosan nanoparticles (CHT-NPs) to mitigate oxidative stress in durum wheat may positively influence the nutritional and technological quality of the grain. In fact, increased polyphenol content in bran was observed in both years of experiments, with CHT-NPs-NAC being the more effective treatment. On the other hand, in 2017, the same treatment had a positive effect on both the tocopherol and tocotrienol content of bran and semolina, while, in 2018, CHT and CHT-NPs were the more effective treatments in increasing the lipophilic antioxidants of bran and semolina. Regarding the technological properties, pasting properties were not altered by any of the treatments, while a tendency towards an enhancement in the gluten aggregation capacity was observed in 2018 in semolina samples. 

Further study is needed to understand the optimum time and concentration for CHT-NPs and CHT-NPs-NAC use, and particularly the effect of environmental conditions on their effectiveness. Although we cannot quantify the benefit of these treatments on the bran and semolina quality, these preliminary results suggest that CHT treatments have the potential to trigger plant defense systems, with a positive effect on grain nutritional quality and with a tendency to induce a technological enhancement in semolina.

## Figures and Tables

**Table 1:** Phenolic acid content (mg/100 g) in bran and semolina obtained from *Triticum durum* (cv. Fabulis) in 2017 and 2018, untreated (Ctrl) or treated with CHT, CHT-NPs, or CHT-NPs-NAC. All data are reported as mean ± standard deviation (*n* = 3) ^a^.

		FA	VA	SYR	CA	p-CA	SA
**2017**	**Bran**						
Ctrl		344.2 ± 25.8 a	8.01 ± 0.06 b	13.30 ± 0.74 a	2.00 ± 0.33	8.01 ± 0.99	35.70 ± 2.61 a
CHT		425.9 ± 23.0 b	8.83 ± 0.02 c	18.06 ± 1.94 b	2.49 ± 0.13	8.83 ± 1.06	43.99 ± 2.81 b
CHT-NPs		386.2 ± 37.5 ab	7.68 ± 0.59 a	12.30 ± 0.52 a	2.20 ± 0.45	7.68 ± 0.89	41.98 ± 2.39 b
CHT-NPs-NAC		414.5 ± 52.3 b	8.97 ± 0.22 d	17.92 ± 3.08 b	1.98 ± 0.50	8.97 ± 0.99	45.72 ± 3.12 b
*p*		*	**	**	*ns*	*ns*	**
	**Semolina**						
Ctrl		28.3 ± 0.8	0.79 ± 0.04	1.79 ± 0.13	0.17 ± 0.04	0.79 ± 0.12 b	5.23 ± 0.43
CHT		28.8 ± 2.5	0.68 ± 0.14	1.54 ± 0.03	0.18 ± 0.06	0.68 ± 0.02 ab	4.96 ± 0.60
CHT-NPs		30.1 ± 3.0	0.68 ± 0.10	1.54 ± 0.29	0.17 ± 0.00	0.68 ± 0.07 ab	5.68 ± 0.75
CHT-NPs-NAC		25.6 ± 1.4	0.59 ± 0.17	1.56 ± 0.28	0.13 ± 0.04	0.59 ± 0.02 a	4.58 ± 0.40
*p*		*ns*	*ns*	*ns*	*ns*	*	*ns*
**2018**	**Bran**						
Ctrl		194.5 ± 7.4 a	4.69 ± 0.33 a	1.77 ± 0.05 a	0.87 ± 0.07	5.81 ± 0.60 ab	24.41 ± 1.13 a
CHT		264.1 ± 25.8 b	5.38 ± 0.47 b	2.51 ± 0.03 b	0.91 ± 0.10	8.38 ± 2.13 b	31.65 ± 3.53 b
CHT-NPs		261.3 ± 3.7 b	5.81 ± 0.50 b	2.27 ± 0.12 ab	0.73 ± 0.21	4.69 ± 1.16 a	30.64 ± 0.68 b
CHT-NPs-NAC		275.9 ± 19.3 b	5.12 ± 0.80 b	2.58 ± 0.25 b	1.02 ± 0.21	5.12 ± 2.34 a	37.08 ± 0.91 c
*p*		*	**	*	*ns*	*	***
	**Semolina**						
Ctrl		26.3 ± 2.7	0.49 ± 0.13 a	0.50 ± 0.06 a	0.11 ± 0.04 ab	0.64 ± 0.03	5.80 ± 0.60
CHT		27.7 ± 2.5	0.52 ± 0.06 b	0.60 ± 0.08 ab	0.05 ± 0.02 a	0.49 ± 0.05	5.76 ± 0.24
CHT-NPs		29.4 ± 6.9	0.50 ± 0.07 a	0.56 ± 0.12 b	0.07 ± 0.02 a	0.50 ± 0.13	6.76 ± 1.09
CHT-NPs-NAC		29.9 ± 4.6	0.64 ± 0.04 c	0.63 ± 0.02 b	0.14 ± 0.06 b	0.52 ± 0.17	6.85 ± 1.11
*p*		*ns*	***	*	*	*ns*	*ns*

^a^ FA, ferulic acid; VA, vanillic acid; SYR, syringic acid; CA, caffeic acid; p-CA, p-coumaric acid; SA, sinapic acid. For each year, asterisks show the significance of treatments in both bran and semolina, following the one-way ANOVA: ***: *p* < 0.001; **: *p* < 0.01; *: *p* < 0.05; *ns*: not significant. Different letters indicate significant differences among treatments according to Tukey’s post-hoc test.

**Table 2:** Tocopherol and tocotrienol content (mg/100 g) in bran and semolina obtained from *Triticum durum* (cv. Fabulis) in 2017 and 2018, untreated (Ctrl) or treated with CHT, CHT-NPs, or CHT-NPs-NAC. All data are reported as mean ± standard deviation (*n* = 3) ^a^.

		(β + γ)-T3	α-T3	(β + γ)-T	α-T	Total T3	Total T
**2017**	**Bran**						
Ctrl		11.85 ± 0.95 b	0.49 ± 0.01	1.40 ± 0.09 a	0.76 ± 0.13	12.33 ± 0.99	2.16 ± 0.04 a
CHT		14.25 ± 0.07 c	0.55 ± 0.06	2.06 ± 0.01 b	1.35 ± 0.00	14.80 ± 0.01	3.41 ± 0.01 bc
CHT-NPs		10.11 ± 1.02 a	0.26 ± 0.01	1.39 ± 0.31 a	1.04 ± 0.23	10.37 ± 1.03	2.43 ± 0.54 ab
CHT-NPs-NAC		12.55 ± 1.15 c	0.29 ± 0.02	2.58 ± 0.38 c	1.58 ± 0.77	12.98 ± 1.17	4.16 ± 1.03 c
*p*		**	*ns*	**	*ns*	*ns*	*
	**Semolina**						
Ctrl		4.35 ± 0.38 a	0.04 ± 0.01	0.68 ± 0.02	0.23 ± 0.02 a	4.39 ± 0.38 a	0.91 ± 0.03 a
CHT		4.02 ± 0.72 a	0.15 ± 0.21	0.63 ± 0.09	0.23 ± 0.02 a	4.17 ± 0.87 a	0.86 ± 0.11 a
CHT-NPs		4.22 ± 0.51 a	0.04 ± 0.01	0.55 ± 0.12	0.30 ± 0.03 b	4.26 ± 0.52 a	0.85 ± 0.14 a
CHT-NPs-NAC		5.50 ± 0.21 b	0.03 ± 0.01	0.78 ± 0.02	0.32 ± 0.01 b	5.53 ± 0.21 b	1.10 ± 0.01 b
*p*		*	*ns*	*ns*	**	*	*
**2018**	**Bran**						
Ctrl		7.53 ± 0.46 a	0.30 ± 0.02 ab	0.42 ± 0.03	0.32 ± 0.03 b	7.83 ± 0.44 a	0.74 ± 0.01 b
CHT		8.26 ± 0.17 b	0.36 ± 0.03 bc	0.50 ± 0.04	0.30 ± 0.04 b	8.62 ± 0.18 b	0.81 ± 0.08 c
CHT-NPs		9.31 ± 0.48 c	0.36 ± 0.01 c	0.48 ± 0.05	0.33 ± 0.02 b	9.67 ± 0.48 c	0.82 ± 0.07 c
CHT-NPs-NAC		8.20 ± 0.31 ab	0.25 ± 0.04 a	0.37 ± 0.01	0.16 ± 0.02 a	8.45 ± 0.35 ab	0.52 ± 0.01 a
*p*		*	**	*	**	*	**
	**Semolina**						
Ctrl		3.50 ± 0.09 a	0.07 ± 0.01 a	0.97 ± 0.05	0.52 ± 0.08 b	3.57 ± 0.09 a	1.49 ± 0.11 b
CHT		4.76 ± 0.05 b	0.07 ± 0.01 ab	1.16 ± 0.08	0.49 ± 0.01 b	4.83 ± 0.06 b	1.66 ± 0.09 c
CHT-NPs		4.29 ± 0.11 b	0.11 ± 0.01 b	0.95 ± 0.05	0.68 ± 0.08 c	4.40 ± 0.11 b	1.64 ± 0.05 c
CHT-NPs-NAC		3.50 ± 0.01 a	0.06 ± 0.02 a	0.88 ± 0.03	0.24 ± 0.10 a	3.56 ± 0.03 a	1.12 ± 0.08 a
*p*		**	*	**	*	**	*

^a^ (β + γ)-T3, (β + γ)-Tocotrienols; α-T3, α-Tocotrienol; (β + γ)-T, (β + γ)-Tocopherols; α-T, α-Tocopherol; Total T3, total tocotrienols; Total-T, total tocopherols. For each year, asterisks show the significance of treatments in both bran and semolina, following the one-way ANOVA: **: *p* < 0.01; *: *p* < 0.05; *ns*: not significant. Different letters indicate significant differences among treatments according to Tukey’s post-hoc test.

**Table 3:** Total and damaged starch (g/100 g db) and pasting properties of semolina obtained from *Triticum durum* (cv. Fabulis) in 2017 and 2018, untreated (Ctrl) or treated with CHT, CHT-NPs, or CHT-NPs-NAC. All data are reported as mean ± standard deviation (*n* = 3) ^a^.

		Total Starch	Damaged Starch	Beginning of Gelatinization	Maximum Viscosity	End of Final Holding Period	Breakdown	Setback
**2017**								
Ctrl		71.34 ± 3.18	11.00 ± 0.52	64.87 ± 0.23	235.67 ± 8.08	650.33 ± 23.29	35.00 ± 1.73	461.00 ± 22.52
CHT		71.71 ± 2.69	11.36 ± 0.08	65.50 ± 0.46	232.00 ± 15.87	641.00 ± 47.03	37.67 ± 2.08	458.67 ± 28.31
CHT-NPs		72.22 ± 4.38	11.01 ± 0.67	65.00 ± 0.52	237.33 ± 10.41	668.67 ± 38.48	35.67 ± 1.53	476.67 ± 29.94
CHT-NPs-NAC		72.76 ± 2.81	11.43 ± 0.42	65.17 ± 0.40	227.33 ± 6.66	643.33 ± 19.35	33.67 ± 2.52	457.33 ± 14.57
*p*		*ns*	*ns*	*ns*	*ns*	*ns*	*ns*	*ns*
**2018**								
Ctrl		67.78 ± 1.77	7.18 ± 0.50	65.46 ± 1.08 ab	183.88 ± 11.86	414.38 ± 25.60	48.86 ± 2.04	285.75 ± 17.98
CHT		67.16 ± 2.21	7.33 ± 0.40	66.19 ± 0.72 ab	176.63 ± 8.94	405.50 ± 13.26	44.50 ± 4.21	277.88 ± 12.16
CHT-NPs		66.99 ± 1.21	7.54 ± 0.51	66.58 ± 1.51 b	169.63 ± 12.37	378.13 ± 31.42	46.63 ± 3.81	259.63 ± 22.92
CHT-NPs-NAC		66.98 ± 0.79	7.34 ± 0.24	64.53 ± 1.34 a	178.00 ± 20.33	401.25 ± 57.81	45.88 ± 5.22	273.25 ± 42.46
*p*		*ns*	*ns*	*	*ns*	*ns*	*ns*	*ns*

^a^ db, dry basis; beginning of gelatinization (°C), temperature at which an initial increase in viscosity occurs; maximum viscosity (BU, Brabender Unit), maximum viscosity reached during the analysis; end of final holding period (BU); breakdown (BU), difference between the maximum viscosity and the viscosity reached at the end of the holding period; setback (BU), difference between the final viscosity and the viscosity reached at the end of the holding period. For each year, asterisks show the significance of treatments in both bran and semolina, following the one-way ANOVA: *: *p* < 0.05; *ns*: not significant. Different letters indicate significant differences among treatments according to Tukey’s post-hoc test.

**Table 4:** Thiols (mg GSH/100 g), total proteins (g/100 g db), and gluten aggregation properties of semolina obtained from *Triticum durum* (cv. Fabulis) in 2017 and 2018, untreated (Ctrl) or treated with CHT, CHT-NPs, or CHT-NPs-NAC. All data are reported as mean ± standard deviation (*n* = 3) ^a^.

		Thiols	Total Proteins	Peak Maximum Time	Maximum Torque
**2017**					
Ctrl		25.14 ± 3.76	13.69 ± 0.68	40.57 ± 2.37	50.91 ± 3.07
CHT		23.05 ± 0.92	13.77 ± 0.38	38.17 ± 2.14	51.80 ± 3.51
CHT-NPs		22.89 ± 2.88	13.33 ± 0.77	44.50 ± 3.73	48.67 ± 3.07
CHT-NPs-NAC		20.72 ± 2.46	13.97 ± 0.23	37.17 ± 1.72	54.50 ± 1.50
*p*		*ns*	*ns*	*ns*	*ns*
**2018**					
Ctrl		18.48 ± 0.88	13.77 ± 0.09	35.56 ± 2.60 a	50.80 ± 1.47 a
CHT		22.81 ± 5.83	13.98 ± 0.05	33.50 ± 2.22 a	52.38 ± 1.12 ab
CHT-NPs		20.66 ± 0.65	14.00 ± 0.22	34.55 ± 2.62 a	53.13 ± 2.38 b
CHT-NPs-NAC		19.01 ± 1.51	13.92 ± 0.27	37.00 ± 2.39 a	52.90 ± 1.56 b
*p*		*ns*	*ns*	*ns*	*

^a^ db, dry basis; peak maximum time, expressed in seconds, corresponds to the peak torque time; maximum torque, expressed in Brabender Equivalents (BE), corresponds to the peak that occurs when gluten aggregates. *: *p* < 0.05; *ns*: not significant. Different letters indicate significant differences among treatments according to Tukey’s post-hoc test.

## Data Availability

Not applicable.

## Supplementary Materials

The following supporting information can be downloaded at: https://www.mdpi.com/article/10.3390/plants11152021/s1, Figure S1: Example of chromatogram acquired by HPLC-DAD (at 280 and 320 nm) on durum wheat bran. See Section 4.2 for details. Peaks: 1, vanillic acid; 2, syringic acid; 3, vanillin; 4, caffeic acid; 5, p-coumaric acid; 6, ferulic acid; 7, sinapic acid; 8, salicylic acid; 9–13, ferulic acid isomers.
