# Supplementary figures and images for: Oxidative Stress Mitigation by Chitosan Nanoparticles in Durum Wheat Also Affects Phytochemicals and Technological Quality of Bran and Semolina
Source: Plants (Basel). 2022 Aug 3;11(15):2021. doi: 10.3390/plants11152021 (PMC9370655; doi:10.3390/plants11152021)

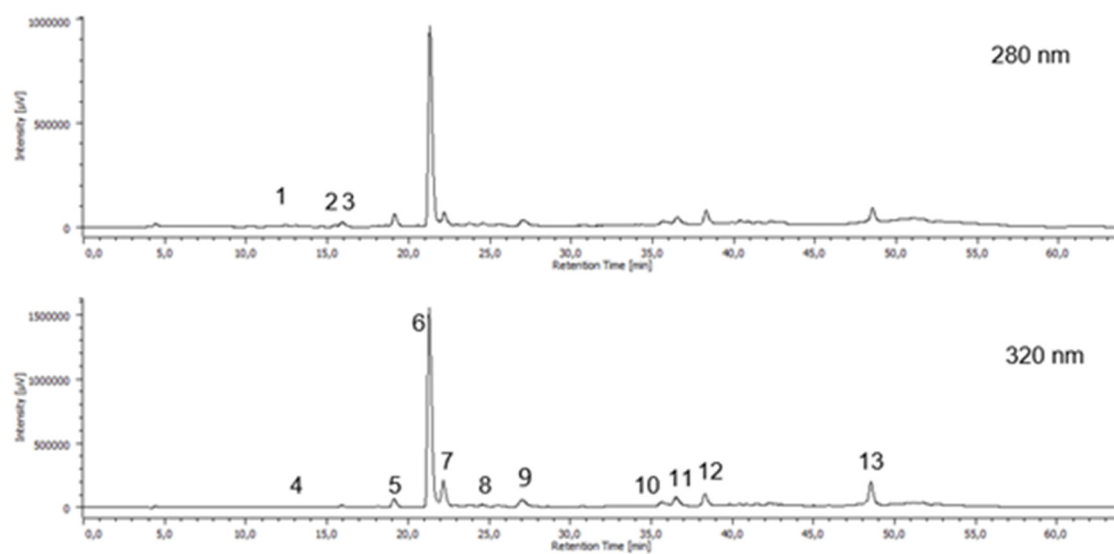

Figure S1. Example of chromatogram acquired by HPLC-DAD (at 280 and 320 nm) on durum wheat bran

Supplement: Supplementary file 1 [file plants-11-02021-s001.zip › plants-1814619-supplementary.pdf]
